# A Treatise on the Diseases of the Bones

**Published:** 1849-12

**Authors:** 


### REVIEWS.

---

ART. V.—*A Treatise on the Diseases of the Bones.* By EDWARD STANLEY, F. R. S., President of the Royal College of Surgeons of England, and Surgeon to St. Bartholomew's Hospital. Philadelphia: Lea & Blanchard. 1849.

The value of clinical instruction is no where more triumphantly vindicated than in the history of St. Bartholomew's Hospital. One of the ablest of the Faculty of that institution has well remarked: "I have always thought that, in hospitals, knowledge is perpetually running to waste, for want of laborers to gather it; and I think so still. I have always thought that, in our schools, every mode of lecturing has been unduly exalted above clinical lecturing; and every place where knowledge is to be had, or supposed to be had, has unduly been preferred to the bedside; and I continue to think thus."

We fully concur with Dr. Latham in these valuable thoughts, and wish they could be properly impressed upon the minds of students of medicine. They cannot spend too much time in the hospital; its fields of instruction are perpetually opening new sources of information that can be gleaned from no other quarter. The next best thing to instruction thus obtained, is that derived from those intelligent minds that have devoted their lives to hospital service, and in this respect the literary efforts of the Faculty of St. Bartholomew's Hospital challenge the respect and confidence of medical students. The literary labors of Abernethy, Blundell, Lawrence, Gooch, and Latham have long commanded the admiration of the profession, and the excellent use they made of their hospital facilities will be remembered while medicine is cultivated as a science.

The work, whose title stands at the head of this review, is another of the rich contributions of St. Bartholomew's Hospital, and is a worthy colleague of the productions to which we have already referred in general terms. A more acceptable offering could not have been made to the medical profession, than this excellent treatise on the diseases of the bones. A philosophical monograph on this subject was needed, and we are thankful that the duty of preparing it devolved upon Mr. Stanley.

In reviews of ordinary works, a general notice is perhaps equal to the demand, but such a treatise as the one before us requires analysis, in order to do justice to its merits, and to convey to the general practitioner those useful and practical details that are valuable in his intercourse with his patients.

When Chesselden published his work on the anatomy of the bones, he apologized for omitting a reference to many of the important bones, by pleading the want of space, and a crusty reader of that day went about enquiring why Chesselden could not have bought a little more paper. The author before us has no apology of the kind to make—his work is almost complete, and presents a gratifying instance of great fullness, in a small compass. In this respect it is even superior to Mr. Brodie's admirable work on the Joints. In the active duties of a general practice, a physician is often at a loss for the very kind of information contained in this volume, and so scattered are the rays of light, that he might search an extensive library of English, French, Italian, and German medical books, without meeting a response to his inquiries. Compared with the production of Mr. Stanley, Mr. Benjamin Bell's Treatise is but a primer.

These facts seem to plead imperatively for an extended analysis of Mr. Stanley's work, and we shall devote all the space we can spare to it. By this course we may supply, partially, the wants of those who cannot procure the work, but we assure our readers that nothing of the kind can be considered a substitute for the treatise itself. The cases, which illustrate the observations, are full of instruction, and should be very carefully studied.

Mr. Stanley opens his treatise with the subject of "Hypertrophy of Bone." The affection, thus designated, consists in an increase of the size of the bone from the augmentation of its healthy tissue. Long bones are often thus increased in thickness, but rarely in length. This rarity, however, constitutes a reason why the practitioner should make himself very familiar with this state of things, for an incorrect diagnosis may lead to serious evil in the way of mal-practice. Mr. Stanley remarks:—"When a bone is hypertrophied only in its length, it may seem that the apparently greater length of it is the consequence of defective growth in the corresponding bone of the opposite limb. But when a bone is increased in thickness, and also in its length, its hypertrophied condition is then distinctly marked. Instances occur of hypertrophy in the tibia, whilst the fibula undergoes no change. Under such circumstances, either the hypertrophied tibia will become curved, or the ligaments uniting it to the fibula will yield with the increase of its length; and I have seen instances of both these occurrences."

It is easy to mistake this state of things for disease of the hip-joint. We have known such mistakes made, but they cannot occur with one who has capacity to take into view all the subject of examination. An intimate knowledge of these things is valuable, for the reason that every thing that tends to a correct diagnosis is important, and hypertrophy of the long bones of the leg may be so readily confounded with some of the hip-joint diseases and the error would be so pernicious, that the practitioner cannot be too vigilant.

The following are the features of hypertrophy of the bone :

"The enlargement of bone by hypertrophy is unaccompanied by pain ; it advances very slowly, its progress often extending through several years. Thickening of the periosteum often accompanies the enlargement of the bones, and ulcerations often occur in the integuments covering them, followed by small exfoliations from the hypertrophied bones." These features show how nearly allied this affection is to scrofulous disease, and hypertrophy of the bone will most usually be found in connection with the scrofulous diathesis.

Hypertrophy of the long bones is considered by Mr. Stanley beyond the reach of either constitutional or local treatment. The local application of iodine, and the internal use of iodide of potassium with sarsaparilla are beneficial to the extent of removing the irritation of the periosteum, but nothing can effect a diminution of the bones.

Hypertrophy occurs in bones of cancellous texture, and frequently in the bones of the face. In the latter, the affection usually begins early in life, and most frequently in the superior maxillary bone. The hypertrophy extends through the whole bone, and into the adjacent bones, causing the enlargement of them, and frequently the obliteration of the cavities of which they constitute the boundaries. The obliteration of the antrum, the nasal passages, and the contraction of the orbit constitute a state of things demanding the attention of the surgeon. Inasmuch as medicine can have no influence upon the disease, the only question to be determined is the propriety of an operation. When this is determined upon, it is not essential to remove the whole of the diseased bone. Mr. Stanley says he knows of instances where only a part of the hypertrophied bone has been removed, and the wound healed soundly over the remaining portion of it, and the operation was not followed by any increase of the disease.

*Atrophy of Bone.*—This affection is a consequence of the diminution of the healthy tissue. "In some instances the bone is merely diminished in size; in others the walls are thinned, and the cells widened, and occasionally the cancellous texture wholly disappears, and the bone after maceration presents the characters of the bone of a bird, with its simple tube and thin walls."

The causes of this state of things are general or local. Atrophy may arise from defective constitutional nourishment, or from a deficiency of the supply of arterial blood to the bone. Again: the want of action, as in a paralyzed limb, is a cause of atrophy. Anchylosis of the shoulder joint is followed by atrophy of the humerus, and anchylosis of the hip joint, by atrophy of the femur.

This general description points out the proper course to the physician. The subject is much less important than that of hypertrophy, and does not therefore deserve much attention.

*Neuralgia of Bone.*—By this term, Mr. Stanley designates a class of cases in which pain arises in a bone, severe and lasting, unaccompanied by inflammation or other organic changes in its tissue, and thus, apparently, constituting a nervous affection of bone, like the neuralgia of other structures.

This species of disease is often met with in practice, and is very intractable. Some of the severest suffering we have ever seen was in cases of neuralgia of the bone. Mr. Stanley says that "cases of it are most apt to occur in females, and are often accompanied by symptoms of hysteria. In the instances which have come under Mr. Stanley's observation, the affection was met most frequently in the condyles of the femur, in the head of the tibia,

4

and in the humerus, and sometimes in the shaft of the bone. In some cases the pain was limited to a small extent of the bone; but in others, the pain extended through the whole of it. Compression of the limb aggravated the pain. There was scarcely any increase of heat in the soft parts covering the bone, which was the seat of the pain, and no more swelling than was occasioned by slight serous effusion into, or thickening of, the subcutaneous cellular tissue.

These observations are not, in all their details, in accordance with our experience. We have seen many cases of this affection, and they occurred more frequently in males than in females, and the os pisiforme has been the seat of the disease more frequently than the condyles of the femur, the head of the tibia or humerus. We have occasionally seen neuralgia in the superior maxillary bone, closely connected with tic doloreux, and when there it may be confounded with inflammation of the antrum, an error that would lead to very bad practice. A very nice discrimination is needed to distinguish the difference between neuralgia and inflammation. The difficulties are not removed by the diagnostic signs mentioned by Dr. Stanley. He says:

"The diagnosis between the neuralgic affection and inflammation of bone must chiefly rest on the following circumstances: first, the character of the constitutional symptoms, as these are, or are not, such as would afford ground for suspecting the local affection to be of a nervous or hysteric nature; second, the character of the local symptoms, *and the influence of remedies upon them.*—The pain attendant on inflammation of bone is usually less severe than the pain of its neuralgic affection; it is rather a constant aching in the bone, than the paroxysms of severe pain, which occur in the neuralgic affection.—Moreover, it is to be observed that local remedies do, although slowly, produce their effect on inflamed bone; they obtain more than a mitigation of the pain; when perseveringly used, they will wholly remove it. Not so in the neuralgic affections, for here the best of remedies obtained but a temporary and partial relief."

This seems to us a singular system of diagnosis. The great end aimed at in diagnosticating diseases is the ap plication of the proper remedies, but according to Mr. Stanley, the remedies themselves, in their failure or success, become a prominent part of the diagnosis. The phenomena attendant upon an inflammatory condition of the bone are sufficiently marked to enable a discriminating practitioner to distinguish that state of things from neuralgia. There are cases in which there is some obscurity, but the two diseases may always be distinguished without the humiliation of experimenting with the treatment, in order to find out what is the matter with the patient. And it is important to remember that the continuance of a neuralgic affection of the bone for any great length of time may produce "changes in the vascular tissue of the bone, that may terminate in sanguineous congestion, and even effusion of blood into it.

Mr. Stanley does not offer any very radiant hopes in the treatment of neuralgia. He says: "Local depletory remedies, sedative applications, counter-irritants, all have obtained only a brief mitigation of the pain; nor have constitutional remedies been more effective, with the exception of opium, which subdued the pain so long as the system was under its influence."

The plan of treatment which we have found most successful, is the application of the vinous tincture of colchicum, with calcined magnesia, in doses of a teaspoonful three times a day, and quinine in doses of ten, fifteen, or even twenty grains two or three times a day. The severest cases we have met have yielded to this treatment.

*Inflammation of Bone.*—Rheumatism, syphilis, and violence are the most fruitful sources of this condition of the bone, and in no one point of pathology, of no graver character than this, is it more important to make a correct diagnosis, than between syphilitic and rheumatic inflammation of bones.

There is an increase in the sensibility, as well as in the vascularity of inflamed bone, and, as Mr. Stanley says, "from the inflamed tissue of bone there are products corresponding with the albuminous products from the inflamed tissue of soft parts. It may be that pus is produced from the inflamed compact osseous tissue. In the inflamed cancellous tissue pus is certainly deposited ; the source of it here, probably, is the fine and very vascular membrane which lines the cells of bone."

"It results from the intimate connexion which, in respect to their vascular and nervous endowments, the component parts of a bone hold one with another, that they readily reciprocate their morbid actions ; not, however, in an irregular manner, for there is an order observable in the progress of inflammatory changes from one part of a bone to another. Inflammation of the medullary membrane is followed by inflammation in the periosteum and outside of the bone. Moderate inflammation of the medullary membrane is followed by thickening of the periosteum, and by osseous deposits on the surface of the bone, with expansion and thickening of its outer lamella. Acute inflammation of the medullary membrane is followed by ulceration of the periosteum, by suppuration beneath it, and by ulceration of the surface of the bone.—Inflammation of the periosteum is followed by thickening of the inner lamella of the bone."

"Enlargement of bone is the general effect of inflammation in its tissues. But its characters vary with the circumstances giving rise to the inflammation; and it may be accompanied by expansion or induration of the bone, or by osseous deposits upon its surface."

These are the prominent phenomena of inflammation of bone, that should be kept in view. An intimate acquaintance with the subject is very valuable in the treatment of syphilitic and rheumatic affections of the bones, and the close observer will find, occasionally, in syphilitic cases an expansion and induration of the bone, accompanied with osseous deposits upon its surface. We have seen cases where an osseous glove was completely formed over the outer surface of the hand; the bones were considerably expanded, and indurated. In these cases the pain was excruciating, and its relief effected no change in the osseous derangement. In the language of Mr. Stanley, "against the tenderness and irritation of the periosteum, which precede and accompany the morbid changes in the bones, treatment may be directed with the best effect, particularly the local application of mercury to the limb, with the administration of iodide of potassium and sarsaparilla, but upon enlarged and indurated bone, medicines have no effect; its condition will be permanent."

The enlargement of bone by osseous deposits on the surface, arises from inflammation of the periosteum. "A gelatinous deposit is first made on the surface of the bone —this deposit becomes cartilaginous, and then osseous. Rheumatic inflammation of the periosteum is probably the most frequent cause of these osseous deposits; but they occur under other circumstances. For instance, in the skulls of young females who have died whilst pregnant, or shortly after parturition, such osseous deposits have been found upon both the outside and the inside of the skull, but more especially in the latter situation."

On the subject of treatment of inflammation of the bone, Mr. Stanley is clear and philosophical. We give way to his remarks:

"Bone is not readily impressed by external agents: its organic changes in health and in disease being of slow progress, the remedies which influence them are proportionably slow in their operation. But, notwithstanding the little susceptibility bone possesses, its nervous and vascular endowments are so far in harmony with the rest of the system, that when the nervous system is unquiet, and the digestive organs are deranged, the disease of bone, like the diseases of the soft parts under the same circumstances, are with difficulty controlled."

For the subdual of acute inflammation of the bone, especially when seated in the medullary membrane of a long bone, the requisite measures must be actively employed. These measures must be directed to the control of vascular action and the removal of pain. "A persistent and severe pain in the centre of a long bone," says Mr. Stanley, "quickly followed by fever and excitement of the nervous system, are sufficient to warrant the apprehension that the medullary membrane is the seat of inflammation, and to call for activity of treatment; for it is most important the disease should be arrested before it advances to the stage of suppuration in the bone, by which the limb and even the life of the patient is likely to be endangered."

The practitioner cannot be too fully alive to the necessity of active measures in cases of this kind. A full survey of all the phenomena of inflammation of bone, and of the probable sequelæ of neglected cases should rouse the physician to the necessity of judicious activity and vigilance, for he cannot easily be more active and industrious than the disease. The remedies that are useful in the inflammation of other structures are useful in this affection of the bone. Leeching will not, of course, subdue inflammation when in a bone, as promptly as it will, when the inflammation is in the soft parts, but it is, nevertheless, called for, and, after a time, will display its good effects. The abstraction of blood, either by leeching, or cupping, from the most eligible portion of the soft parts, in the region of the affected bone, and fomentations made with cloths, dipped in hot water, and, after being applied to the inflamed part, wrapped in dry cloths, are the appropriate local measures. The position of the inflamed bone must not be neglected—it must be such as will neither invite nor encourage a sluggish circulation, or a congestion in the part, and perfect rest must be enjoined. Mr. Stanley also recommends, as "a local remedy of much value for subduing inflammation in bone, mercurial ointment constantly and plentifully applied to the surrounding parts." It is a remedy, however, that requires much judgment in its use, and must be narrowly watched. We are better pleased with Mr. Stanley's recommendation of iodide of potassium. He says he has found as much benefit from small doses as from large ones; he advises from two to three grains three times a day, in camphor mixture, or in a vegetable bitter infusion. The reader will find, in the department of selections, in this number, a more eligible mode of administering the iodine than either of these methods, and we are satisfied that the constitutional effects of the iodine, when given this way, will be much more useful and permanent. We allude to M. Marchal's preparation of the iodine in fresh almond oil, dissolved at the time of using it.

It is in the inflammation of membranous structures that the iodide of potassium has the best influence, and its good effects are most likely to show themselves when the seat of inflammation is the periosteum or medullary membrane, rather than the osseous tissue. "But," as Mr. Stanley well observes, "the inflammatory action, whatever was its original seat, soon spreads through the entire bone; therefore iodide of potassium is the remedy for inflammation in bone under all the circumstances of its occurrence."

We fully concur with Mr. Stanley in the following statement: "My experience of the iodide of potassium in the syphilitic affections of bone, agrees with the records on this subject by Martin Hassing, of Copenhagen, who states that, 'in no other symptom of syphilis is the iodide so efficacious, and its effects so certain as in these cases of pain in the osseous system, whether they occur by night of day, or have troubled the patient for years, or only for a few days;' and he subjoins a statistical account of seventy-three cases of pains of the bone treated by iodide of potassium, in sixty-five of which the pains wholly disappeared, while in three they were diminished, and in five the iodide was given without effect."

We should rejoice if reporters of statistical facts in medicine would remember the importance of full details. The numerical method, in order to be useful, must not be confined to a mere "numbering of the host;" but should embrace everything that can serve to throw light upon the special condition of each patient. The age, sex, employment, habits, hereditary diathesis, &c., &c., are of the last importance, and the omission of such facts, renders the numerical method nearly as useless, as the loose, undefined plan of taking up stray facts, and wondering over them, which obtains too extensively. Practical medicine will be relieved of much rubbish whenever the true method of enumerating cases comes into universal use, and a simple, reliable and successful Materia Medica will be one of its boons.

But to return—the iodide of potassium will rarely disappoint the practitioner, in its administration in the secondary forms of syphilis. A long experience in its use enables us to speak, with much confidence, of its value in those affections, whether they show themselves in the fibrous or osseous tissues.

In the acute affections of bone, when seated in the medullary membrane, iodide of potassium is inadmissible, and Mr. Stanley very properly recommends the internal use of mercury, in combination with its external employment. These cases are, however, rare, "in comparison with those affections of the bone, for which iodide of potassium is the suitable and almost invariably effective remedy."

Mr. Stanley speaks very commendably of the good effects of counter-irritation, in "long-enduring, or often repeated inflammatory affection of the bone." When counter-irritation is resorted to, it must be kept in full activity. And it must be remembered that counter-irritation is applicable to all the painful enlargements, indurations, and thickenings of bone, which other remedies have failed to relieve. We can fully corroborate these statements —for the results of counter-irritation, in inflammatory conditions of bone, have often come under our observation. In addition to the above admonitions, Mr. Stanley gives the following, which deserve attention. "Counter-irritation must not be employed whilst the inflammatory processes in the soft parts around the diseased bone are active; nor until they have subsided to allow an issue or seton to be placed near enough to the diseased bone to control the action within it. The degree of thickness of the soft parts over the diseased bone, must determine the extent and situation of the counter-irritant. When the carpal or tarsal bones are the seat of disease, a large issue placed directly over them will be likely, from the thinness of their investing soft parts, to aggravate the disease. When, on the other hand, the bones of the hip, or of the shoulder joint, are the seat of the disease, the thickness of the investing soft parts permits the issue to be placed directly over them. The counter-irritation is a controlable disease, established on the surface of the limb, in order to divert the activity of the organic processes from the diseased bone; but that it may have this effect, care must be taken that the new disease does not extend its sphere of irritation to the old one. Where suppuration is going on from the soft parts around the diseased bone, counter-irritation is inexpedient, for if a free outlet is given to the matter, all the good effects of the issue or seton will be obtained. This is true in all cases, except where the discharge is trifling—in such cases counter-irritants are not interdicted.

We are reluctantly compelled to close the analysis of this admirable work, at the very threshold of some of the most important subjects. In many points of view, we regard Mr. Stanley's "Treatise on the Diseases of the Bones," as the most valuable contribution to Surgery we have seen for years, and we desire to make our readers as familiar with its revelations as a full analysis can make them. To the fulfilment of this purpose we shall address ourselves in the January number.

